# AIDS in the Brazilian Amazon: epidemiological trends and disparities across states

**DOI:** 10.3389/fpubh.2025.1631596

**Published:** 2025-12-01

**Authors:** Thiago Augusto Ferreira dos Anjos, Ana Paula Ferreira David, Ruth Stephany Costa Silva, Sebastião Kauã de Sousa Bispo, Bruna Labibe Amin da Silva, Adson Lucas Ferreira de Almeida, Luiza Raquel Tapajós Figueira, Marcos Jessé Abrahão Silva, Daniele Melo Sardinha, Lilian Cristina Santos sinfronio da Silva, Rebecca Lobato Marinho, Everaldina Cordeiro dos Santos, Luana Nepomuceno Gondim Costa Lima

**Affiliations:** 1 Program of Epidemiology and Public Health Surveillance of the Evandro Chagas Institute (PPGEVS/IEC/MS), School of Public Health of the University of São Paulo (FSP/USP), São Paulo, Brazil; 2 University of the Amazon (UNAMA), Ananindeua, Brazil; 3 Bacteriologic Section – Evandro Chagas Institute (IEC), Ananindeua, Brazil; 4 Postgraduate Program in Parasitic Biology in the Amazon, State University of Pará and Evandro Chagas Institute (PPGBPA/UEPA/IEC), Belém, Brazil; 5 Program of Epidemiology and Public Health Surveillance of the Evandro Chagas Institute (PPGEVS/IEC/MS), Ananindeua, Brazil; 6 Bacteriology and Mycology Section, Molecular Biology Laboratory, Evandro Chagas Institute (SABMI/LABMOL/IEC), Ananindeua, Brazil; 7 Postgraduate Program in Epidemiology and Health Surveillance, Evandro Chagas Institute (PPGEVS/IEC), Ananindeua, Brazil

**Keywords:** epidemiology, public health surveillance, indicators, AIDS, programming languages

## Abstract

Acquired Immunodeficiency Syndrome (AIDS), caused by the Human Immunodeficiency Virus (HIV), remains a major public health problem in Brazil. Infection rates vary greatly between regions and states. The North region, in particular, has a higher number of cases, making it a long-lasting challenge, especially in a region with many social and economic challenges. This ecological, descriptive, and analytical study examined AIDS trends across Northern Brazilian states from 2013 to 2023 using data from Brazil’s Notifiable Diseases Information System (SINAN), Mortality Information System (SIM) and national HIV/AIDS epidemiological reports. Our R-based analytical approach incorporated descriptive statistics, Joinpoint regression, linear modeling (calculating trend coefficients, determination coefficients, and *p*-values with significance at *p* < 0.05), plus heatmap clustering with dendrograms to evaluate inter-state rate patterns. Spatial variation analysis revealed distinct epidemiological patterns: four states (Amazonas, Amapá, Tocantins, and Rondônia) showed declining detection rates, while Acre experienced a concerning >90% increase despite stable mortality rates. These findings emphasize important groups of cases and identify which states should prioritize public health efforts. This information can assist in more effectively allocating resources to areas with the most cases.

## 1 Introduction

Acquired Immunodeficiency Syndrome (AIDS), caused by the Human Immunodeficiency Virus (HIV), remains a major global challenge, particularly in low- and middle-income developing countries. Brazil has been a pioneer in HIV elimination and control policies since the 1980s, providing free antiretroviral drugs (ARVs), sexual education, awareness campaigns, and programs for vulnerable populations. These initiatives have enabled Brazil to maintain disease control in some regions in recent years. However, the country still faces challenges, particularly in certain areas with fluctuating detection rates of new cases (1, 2).

Within the current epidemiological context, Brazil has a significant number of people living with HIV/AIDS distributed across different regions. According to the most recent data from the Ministry of Health, an estimated 960,000 people live with HIV in Brazil, with an estimated prevalence of 0.4% in the general population. However, this national average masks significant regional disparities, with some areas showing higher rates, suggesting the need for tailored regional health policies to address location-specific challenges. Among regions with the highest infection rates, the Northern region stands out (3–5).

The Northern region, historically characterized by geographical and socioeconomic challenges that hinder healthcare access, has shown concerning trends in new HIV/AIDS cases. Epidemiological data reveal that this region has experienced some of the highest diagnosis growth rates in the past 5 years, sometimes exceeding national detection averages. This growth stems from multiple factors, including inadequate prevention and treatment infrastructure, limited access to information, and social disparities that increase vulnerability among specific population groups such as youth, indigenous communities, and riverine populations. Furthermore, the limited availability of specialized services and insufficient local prevention campaigns have exacerbated the situation (6, 7).

Understanding the reasons behind the differences in AIDS cases in the North region is crucial. We should also examine how the disease has evolved across the different states over the past decade. This can help us identify any hidden problems or gaps that are influencing the current pattern of the disease.

## 2 Methods

### 2.1 Type of study

This is an ecological study with descriptive, analytical, and demographic characteristics of reported AIDS data from the Notifiable Diseases Information System (SINAN), the Mortality Information System (SIM), and Ministry of Health databases available on the Informatics Department of the Unified Health System (DATASUS) website (DATASUS—Ministério da Saúde), which publishes information on various health conditions, clinical and epidemiological data on Brazil’s health situation.

### 2.2 Data collection site and population

**Figure 1:**
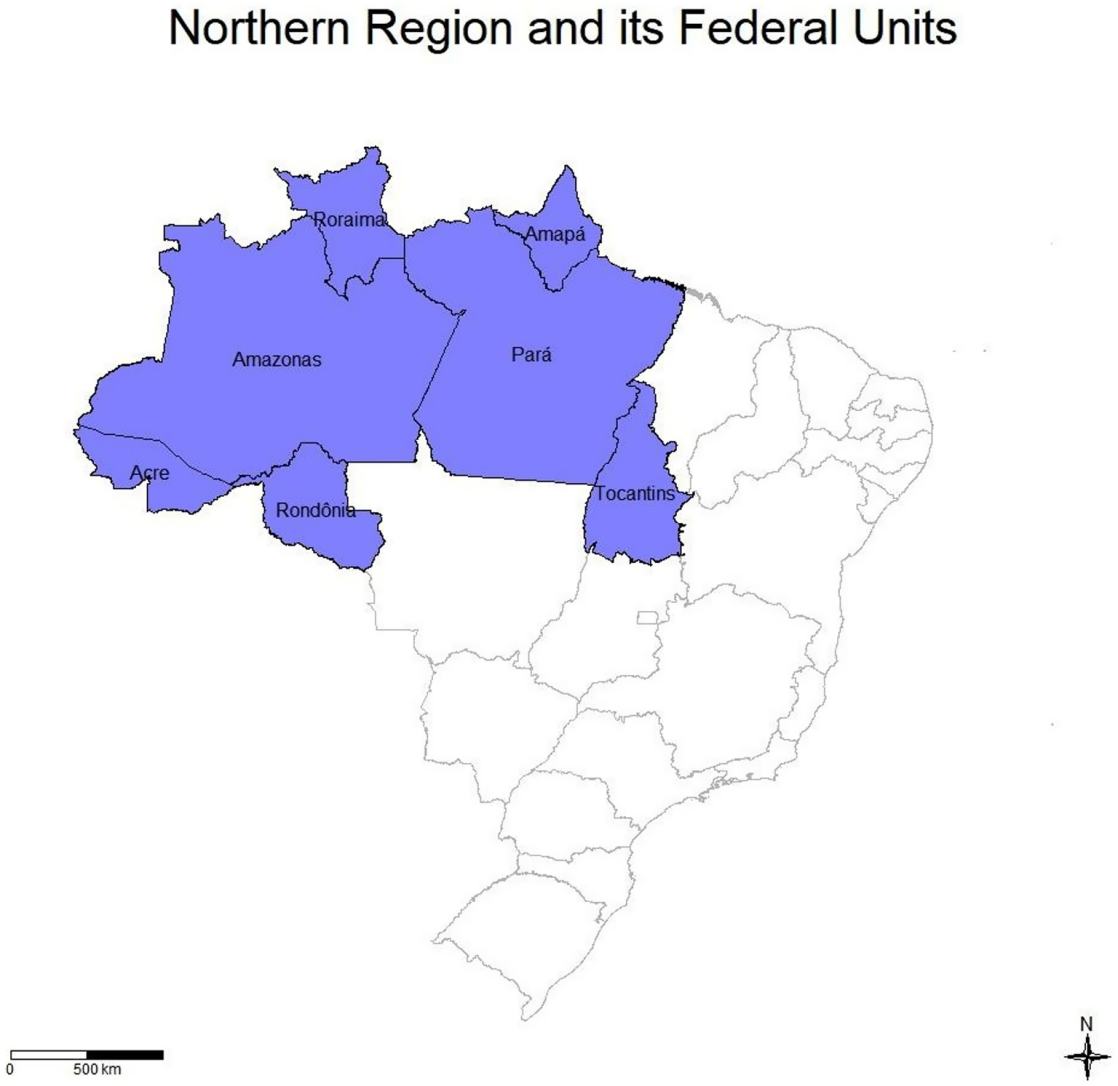
Division of the North region and its federal units, also showing the geographical delimitation of each one. Source: dos Anjos et al. (21).

This research is part of a broader study on Brazil, where researchers identified significant changes in the Northern Region, one of the areas with the greatest socioeconomic and demographic precariousness in the country. The region comprises seven federal units: Acre, in the far west, bordering Bolivia and Peru; Amapá, located in the far north and crossed by the Equator; Amazonas, in the center of the region and Brazil’s largest state by territorial extension; Pará, situated in the eastern zone and the most populous state in the region with 8,120,131 million inhabitants; Roraima, in the far north, bordering Venezuela and Guyana; Rondônia, in the southwest, bordering Bolivia; and Tocantins, located in the southwest portion (Figure 1). According to 2022 data from the Brazilian Institute of Geography and Statistics (IBGE), the region’s total population was approximately 17,354,884 people, with a demographic density of 4.51 inhabitants/km^2^ (8).

### 2.3 Tabulation and research variables

As this study is part of a broader research project, data tabulation was a critical step to ensure the integrity and reliability of the dataset. This process involved organizing the information systematically and removing duplicate or incomplete records that could introduce selection bias and compromise the accuracy of the analysis. The variables considered were: reported AIDS cases in the Northern Region, detection rate, and standardized mortality rate. For this study, the time unit used was year, with data aggregated annually from 2013 to 2023. This decision was based on the structure of the official databases consulted, such as Information System for Notifiable Diseases (SINAN) and Mortality Information System (SIM), and HIV/AIDS monitoring websites, where the collected data were organized by year. The annual aggregation of the data allowed for consistent trend analysis across the North region and its federative units, facilitating comparisons and ensuring methodological consistency throughout the study.

### 2.4 Formulas used in the study

Formula for the AIDS detection rate, made per 100,000 inhabitants:


$$\text{Detection Rate}=(\frac{\begin{array}{c} \text{Number of}\;\mathrm{new}\;\text{AIDS}\;\\ \text{cases in the period} \end{array}}{\text{Total Population the period}})\times 100,000$$

Formula for the mortality rate, made per 100,000 inhabitants:


$$\text{Mortality Rate}=(\frac{\begin{array}{c} \text{Number of}\;\mathrm{new}\;\text{AIDS}‐\text{related}\;\\ \text{deaths in the period} \end{array}}{\text{Total Population the period}})\times 100,000$$

Formula for the variation of cases, made by the percentage of 100:


$$\text{Percent variation}(\%)=(\frac{\text{Final Rate}‐\text{Intial Rate}}{\text{Intial Rate}})\times 100$$

### 2.5 Statistical analysis

For data analysis, R software was used, utilizing violin plots with built-in boxplots that provided information on case concentration, distribution, mean, median, and quartiles for the period 2013–2023. In our analysis, we applied the type 6 parameter of the R quantile function. Although nine different methods are available, type 6 is one of the most widely used in epidemiological studies. It is worth noting that the calculation of the mean, median, and quartiles may have presented slight variations due to rounding to one decimal place, which does not compromise the interpretation of the violin plots or their embedded boxplots.

Regarding the representation of time on the x-axis in the violin plot, although the years are not explicitly labeled, the data cover the period from 2013 to 2023. The visualization structure was designed to prioritize statistical comparison between states, with cases aggregated by state over the 11-year period. This approach allowed for more robust inferences about the quantitative concentration of records, highlighting measures of central tendency, such as mean, median, and quartiles (Q1 and Q3). The decision not to represent each year individually was intended to avoid data fragmentation and increase the clarity of statistical parameters.

The Joinpoint model was applied to identify the years in which the trend changed and how the trend remained after identifying the year in which the change occurred. The blue line is the trend, the red dots are the actual years of the values and the green dot is the Joinpoint identifying the year of the change, making it one of the first studies to use this methodology in the northern region of Brazil.

Linear regression was used to assess the temporal trend in cases, calculating the trend coefficient (annual variation in cases), the coefficient of determination (R^2^, considered significant when ≥0.50) and the *p*-value to confirm statistical significance, *p*-values <0.05 were significant to confirm this trend. In the regression line, there were adjustments with 95% confidence intervals, where the proximity between the line and its limits reflects the accuracy of the analysis.

Two spatial analysis approaches were used in this study. The first was a heatmap combined with a dendrogram, which allowed the identification of patterns of similarity between states and years based on detection and mortality rates. The dendrogram is especially valuable because it organizes the data into clusters, making it easier to visualize which states and time periods share similar epidemiological behaviors. Unlike a simple chronological arrangement, this method highlights structural and contextual similarities that might not be evident otherwise, helping to guide more targeted public health strategies. The second approach was a spatial–temporal variation analysis, which calculated the percentage change in detection rates between 2013 and 2023 using the formula [(2023–2013)/2013 × 100]. This is a standard method in epidemiological studies and was used to identify which states experienced increases or decreases in cases over the 10-year period.

Two spatial analysis approaches were carried out: (1) heatmap with dendrogram that identified patterns of similarity between states and years (with years not ordered for better visualization of the groupings, as it identified which years and states showed these similarities in the detection and mortality rate), and (2) spatial temporal variation that calculated the percentage variation between 2013 and 2023 [(2023–2013)/2013 × 100], a standard method in epidemiological studies, showing which states showed an increase or decrease in cases.

### 2.6 Ethics committee

Due to the fact that the information was in the public domain, on the Ministry of Health’s websites, without manipulation and access to population-specific information, the study did not need to be approved by the ethics committee for research on human beings, as these types of studies are supported by resolution 510 of 2016 (9).

## 3 Results

Between 2013 and 2023, the state of Pará saw an increase in the number of cases, rising from 1.876 to 2.301. Amazonas saw a slight reduction, from 1.420 to 1.386, and Rondônia fell from 454 to 403 cases. Although Acre and Roraima have smaller absolute numbers, both states showed significant proportional growth over the decade. This trend is concerning, especially when we look at population-adjusted rates (Table 1).

**Table 1:** Absolute number of aids cases in the North Region and its federative units, between 2013 and 2023.

Aids cases	2013	2014	2015	2016	2017	2018	2019	2020	2021	2022	2023
Norte	4.437	4.572	4.364	4.482	4.199	4.615	4.817	3.652	4.896	4.916	4.992
Acre	67	73	60	69	71	104	76	45	103	115	144
Amapá	203	187	150	225	238	220	198	170	217	220	211
Amazonas	1.420	1.543	1.214	1.195	1.078	1.177	1.403	1.192	1.671	1.390	1.386
Pará	1.876	1.975	2.187	2.273	1.994	2.305	2.405	1.663	2.146	2.343	2.301
Rondônia	454	426	365	331	379	341	318	279	351	384	403
Roraima	158	147	160	184	188	239	240	133	185	232	271
Tocantins	259	221	228	205	251	229	177	170	223	232	276

Data collected, adapted and analyzed from the Ministry of Health, 2025.

In the AIDS detection rate, there were fluctuations in the federal units, with some states showing growth and others small reductions. In the Northern region in 2013 the rate was 26.1 compared to 26.4 in 2023; Acre was 8.3 (2013) and 15.9 in 2023; Amapá decreased with 27.3 (2013) and 24 in 2023, Amazonas also declined with 37.9 (2013) and 32.5 (2023), Pará showed 23.3 (2013) and 26.2 (2023), Rondônia with decrease 27.4 (2013) and 22.2 (2023); Roraima significant increase with 32.2 (2013) and 41.5 (2023) and Tocantins at 17.6 (2013) and 17.2 in 2023, as shown in Table 2.

**Table 2:** AIDS detection rate per 100.000 inhabitants in the states of the Northern Region, 2013–2023.

Detection rate	2013	2014	2015	2016	2017	2018	2019	2020	2021	2022	2023
Norte	26.1	26.6	25	25.3	23.4	25.4	26.1	19.6	25.9	26	26.4
Acre	8.3	8.9	7.2	8.2	8.3	12	8.6	5	11.4	12.7	15.9
Amapá	27.3	24.5	19.2	28.3	29.3	26.5	23.4	19.7	24.7	25.1	24
Amazonas	37.9	40.4	31.2	30.2	26.8	28.8	33.9	28.3	39.1	32.6	32.5
Pará	23.3	24.2	26.5	27.3	23.7	27.1	28	19.1	24.4	26.7	26.2
Rondônia	27.4	25.4	21.5	19.3	21.8	19.4	17.9	15.5	19.3	21.2	22.2
Roraima	32.2	29.3	31.2	35	34.4	41.5	39.6	21.1	28.3	35.5	41.5
Tocantins	17.6	14.9	15.2	13.5	16.3	14.7	11.3	10.7	13.9	14.4	17.2

Data collected, adapted and analyzed from the Ministry of Health, 2025.

The AIDS mortality data showed distinct patterns across all federal units, with Roraima displaying significant growth from 6.4 (2013) to 7.7 (2023), and Rondônia showing slight growth from 4.9 (2013) to 5.0 (2023). This contrasted with other states that exhibited minor reductions: Pará from 7.9 (2013) to 6.9 (2023), Amazonas from 8.7 (2013) to 6.9 (2023), and Tocantins from 3.8 (2013) to 2.5 (2023). Meanwhile, Amapá showed a significant decrease from 8.2 (2013) to 5.9 in 2023, while Acre maintained relative case stability, as shown in Table 3.

**Table 3:** AIDS mortality rate per 100,000 population in the states of the Northern Region, 2013–2023.

Mortality	2013	2014	2015	2016	2017	2018	2019	2020	2021	2022	2023
Norte	7.1	7.3	6.6	7.1	6.6	6.4	6.1	5.9	6.4	6.2	6.1
Acre	2.3	2.7	1.4	2.6	3.4	3.6	2.2	2.3	2.6	1.3	2.3
Amapá	8.2	7.8	4	5.1	5.3	4.6	5.9	6.4	5.1	6.1	5.9
Amazonas	8.7	8.8	7.6	8.9	7.5	7.1	6.4	6.7	7.7	6.9	6.9
Pará	7.9	8.2	8	8	7.6	7.7	7.8	7	7.2	7.2	6.9
Rondônia	4.9	4.8	4.4	5.1	5.9	3.6	3.1	3	5	3.9	5
Roraima	6.4	7.3	6.9	7.7	4.8	7.8	5.8	5.1	6.4	6.5	7.7
Tocantins	3.8	4.3	3.3	3.5	3.2	3.3	2.6	3	3.5	3.8	2.5

Data collected, adapted and analyzed from the Ministry of Health, 2025.

**Graph 1:**
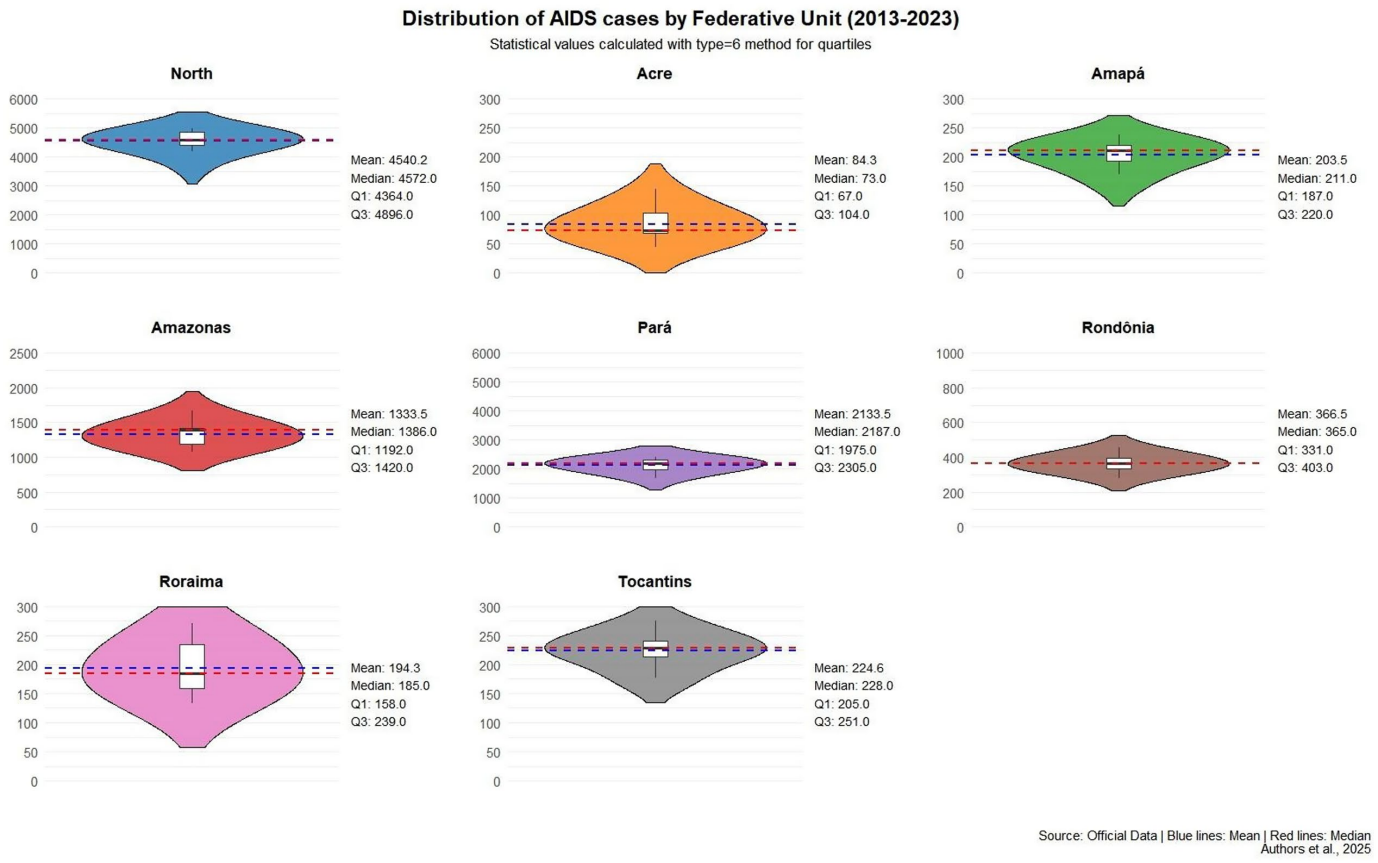
Violin plot statistics for AIDS cases in the Northern region from 2013 to 2023. The width of each violin indicates the concentration of cases during a given period; wider sections reflect higher concentrations. For instance, the Northern region shows a concentration between 3.000 and 5.500 cases. Inside each violin, box plots display the mean, median, and quartile values. Source: Data collected, adapted and analyzed from the Ministry of Health, 2025. *The values of mean, median, and quartiles may vary according to the quartile calculation method (specified as type = 6), but this is expected and does not invalidate the analysis, as the method is consistent. In other words, whether the values of mean, median, and quartiles are slightly higher or lower, it does not invalidate the descriptive information.

Graph 1 presents violin plots combined with box plots to illustrate the distribution of AIDS cases across the Federative Units of Brazil’s North region from 2013 to 2023. The wider sections of each violin indicate periods with higher concentrations of cases. The box plots embedded within each violin show key statistics such as the mean (blue line), median (red line), and quartiles, making it easier to compare states. The data reveals that Pará, Amazonas, and Rondônia consistently had the highest case concentrations. For example, Pará had a mean of 2,133.5 cases and a median of 2,187.0, while Amazonas and Rondônia followed with lower but still significant values. These visualizations help highlight regional disparities and trends over time, supporting targeted public health strategies.

**Graph 2:**
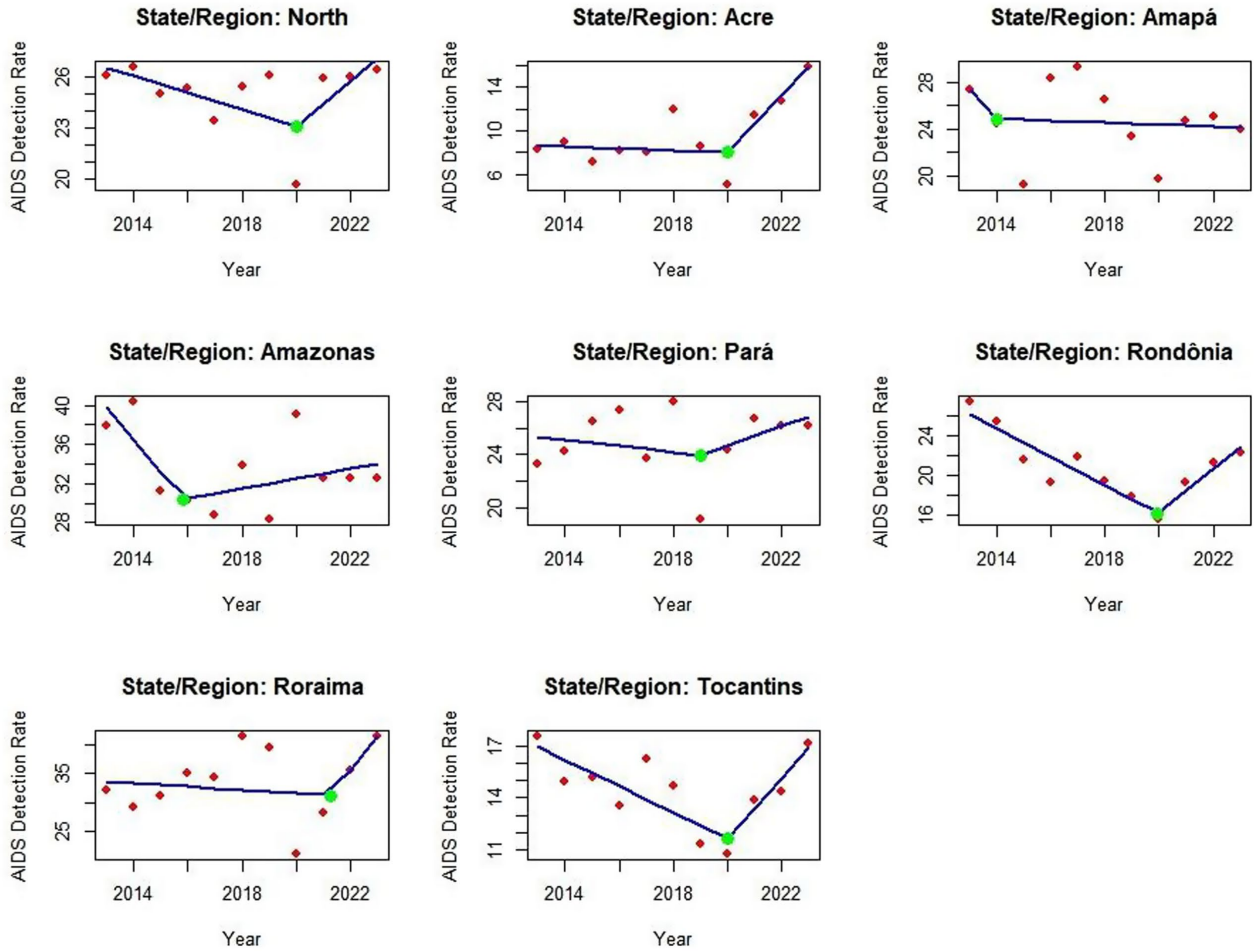
Junction point regression graph showing trends in AIDS detection rates in the Northern region of Brazil from 2013 to 2023. Red dots represent annual detection rates per 100.000 population, while green markers indicate statistically significant changes in the trend. The model highlights key inflection points in the trajectory of the worsening factor. Source: dos Anjos et al. (21). With data adapted from the Ministry of Health.

The Joinpoint analysis model provided essential mapping for the research, showing in each federal unit where these trend changes occurred - whether for growth, stagnation, or reduction. The blue line shows the trend, the red points show the actual detection rate values, and the green point is the Joinpoint indicating where the change in AIDS detection rates occurred. The analysis revealed diverse changes across various states. For instance, in the Northern region itself, the Joinpoint (green point) indicated a trend change in 2020, during the Covid-19 pandemic. Similar changes occurred in 2020 for Acre, Pará, Rondônia, and Tocantins. Other states showed changes in different years, such as Amapá where the Joinpoint indicated a reduction trend since 2014, as shown in Graph 2, which describes the situation in the region and its federal units (Graph 2).

**Map 1:**
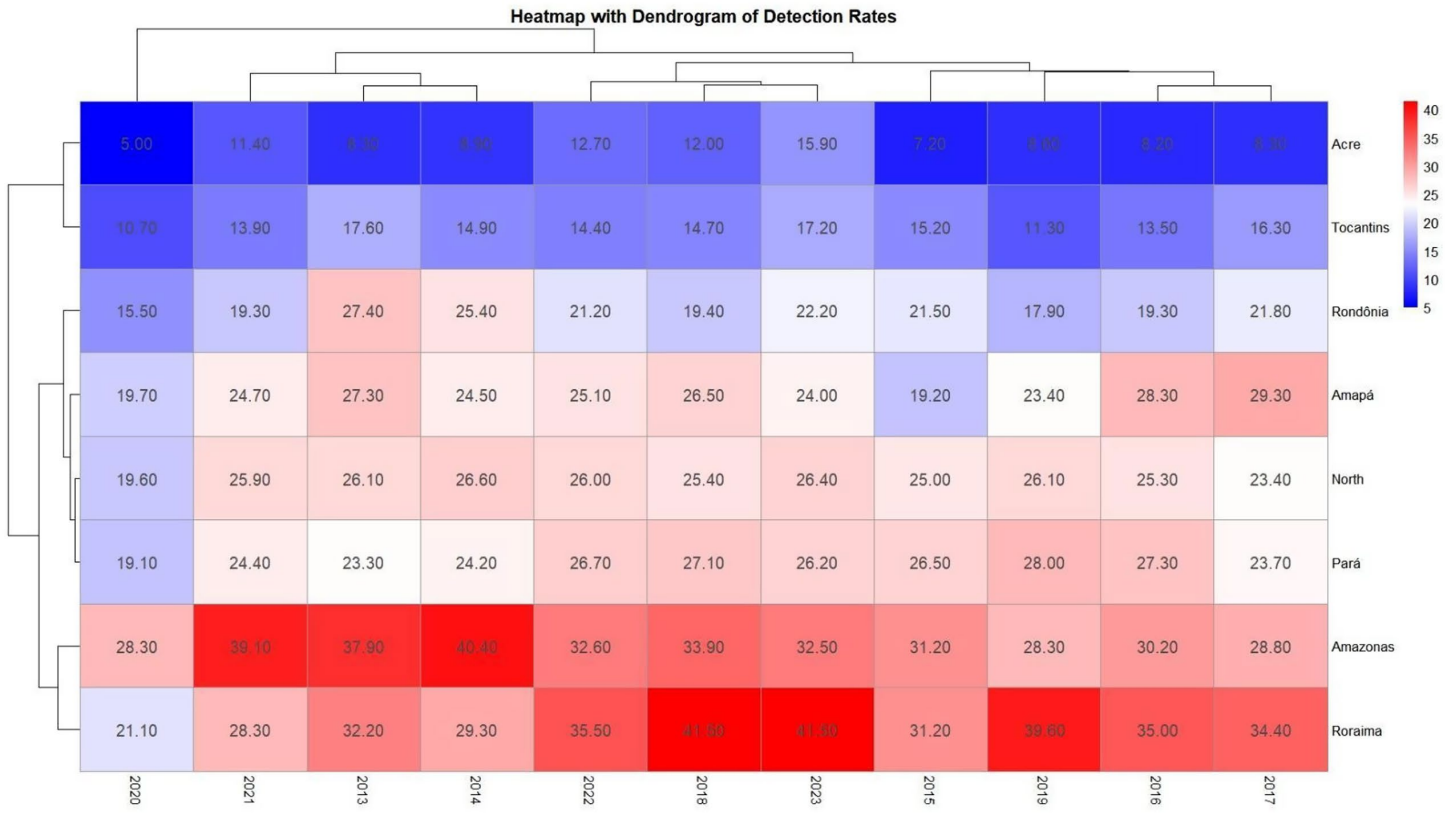
Heatmap comparing AIDS detection rates by year and federal unit, accompanied by a dendrogram (cluster tree) to visualize similarity levels. The vertical axis represents temporal similarities across years, while the horizontal axis compares federal units, including the Northern region. This visualization supports the identification of clusters and patterns in detection rates over time. Source: dos Anjos et al. (21). Data adapted from the Ministry of Health.

The creation of a heatmap, using detection rate values, revealed which years and federal units showed similarity in detection rates, identifying states with comparable patterns. The dendrogram or cluster tree associated units and years with similarities. Note that the years are not in numerical order, precisely because the mapping was designed to group similarities by year—for example, in 2015, 2016, 2017, and 2019 all states showed rate similarities (vertical axis). In 2018, 2022, and 2023, case similarities were very alike. On the horizontal axis, Amazonas and Roraima showed high similarity, while Pará, Amapá, and Rondônia clustered with the study’s reference (the Northern Region); Acre and Tocantins demonstrated similarity across the entire timeframe, as described in Map 1

**Graph 3:**
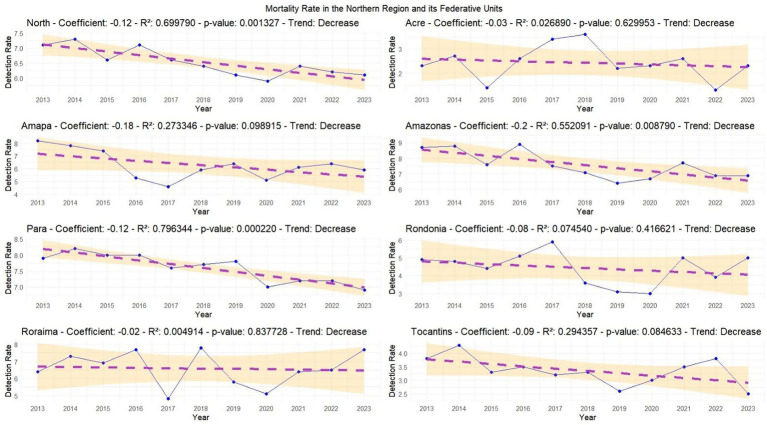
Regression analysis of AIDS mortality rates in the Northern region and its federal units. The yellow band represents the 95% confidence interval, indicating the reliability of the data. A lower *p*-value (below 0.05) suggests statistically significant trends. The proximity of data points to the regression line reflects the accuracy of the model. Source: dos Anjos et al. (21). Data adapted from the Ministry of Health.

In the mortality regression analysis, the Northern Region (*p*-value: 0.001327) and the states of Amazonas (*p*-value: 0.008790) and Pará (*p*-value: 0.000220) showed significant reductions between 2013 and 2023, where R2 was greater than 50% and the *p*-value was below 0.05, indicating significant results confirming a downward trend in mortality rates. For the other states—although the trend suggests reductions in Amapá, Acre, Rondônia, Roraima and Tocantins—the *p*-value was not significant enough to confirm this result (Graph 3).

**Graph 4:**
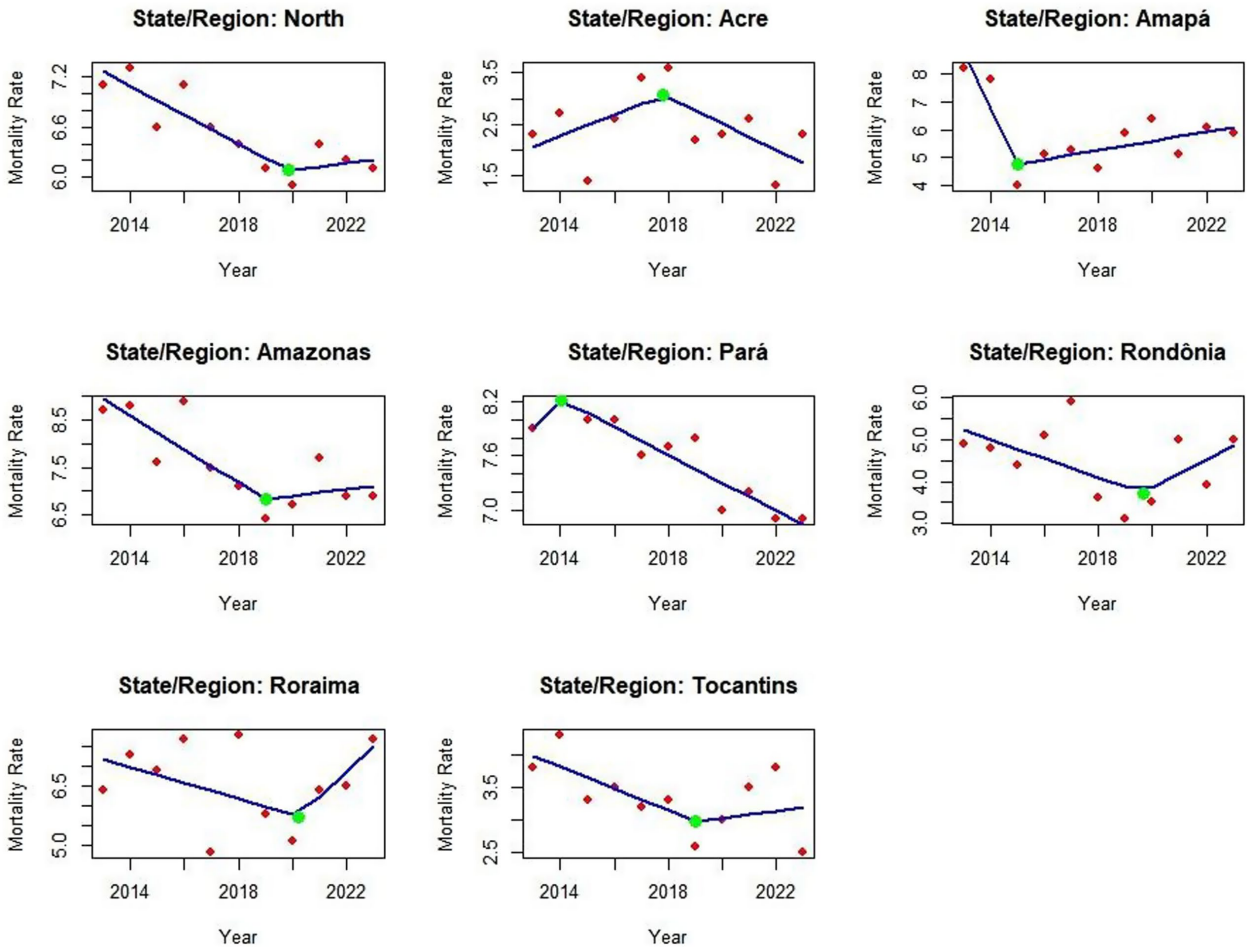
Joinpoint regression analysis of AIDS mortality rates in the Northern region and its federal units from 2013 to 2023. The model displays trend changes over time, with each segment representing a statistically distinct period. This visualization supports the identification of temporal shifts and regional patterns in mortality rates. Source: dos Anjos et al. (21). Data adapted from the Ministry of Health.

In the mortality rate analysis, the Joinpoint regression showed reductions in nearly all variables except Roraima, which displayed an increase following the Covid-19 pandemic. Pará state has demonstrated a consistent decline in mortality cases since 2014, while in Amazonas the green point (Joinpoint) indicated this reduction began in 2019. For Rondônia and Roraima, this mortality trend started increasing in 2020. Notably, Acre experienced its highest mortality peak in 2018, after which the Joinpoint showed a decline. In Tocantins, a mortality rate reduction was observed in 2019, as illustrated in Graph 4.

**Map 2:**
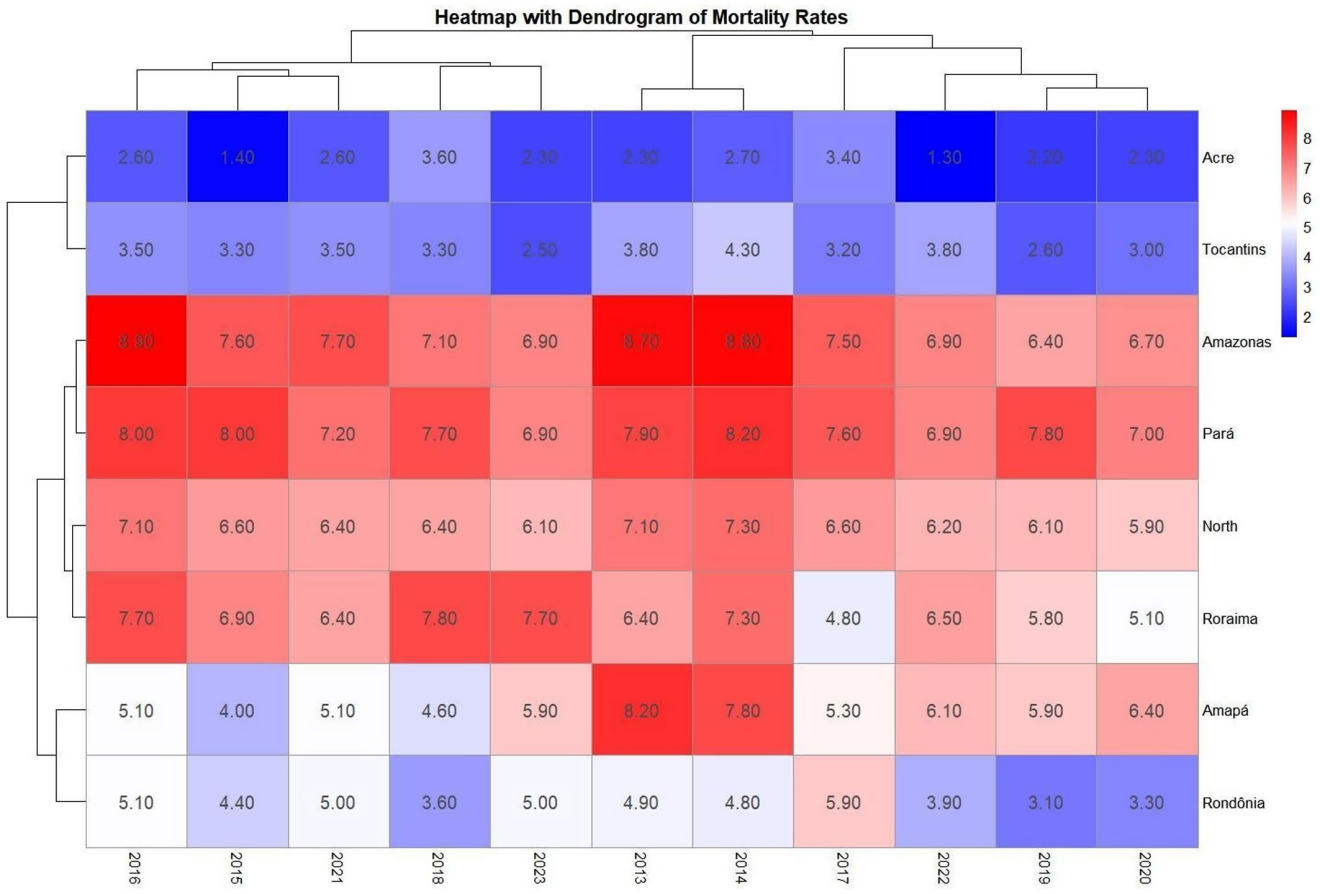
Combined visualization of AIDS mortality patterns in Brazil’s Northern region from 2013 to 2023. The dendrogram highlights relationships among states (horizontal axis) and shifts over time (vertical axis). Color gradients and branching structures help reveal shared trends and temporal divergences across federal units. Source: dos Anjos et al. (21). Data adapted from the Ministry of Health.

The heatmap of mortality coefficients revealed years and units with similar AIDS mortality patterns. Pará and Amazonas showed consistent similarities from 2013 to 2023 across multiple years, exceeding the Northern Region’s mortality coefficient. While Roraima’s pattern resembled the Northern Region’s—a concerning finding—Acre and Tocantins maintained mortality coefficients between 2.00 and 3.00, demonstrating similar 10-year trends. Red coloration indicates high rates. The dendrogram heatmap displays temporal and spatial similarities, explaining why years appear non-sequentially: red represents high rates, white signifies low rates, and blue indicates values below 5 (Map 2).

**Map 3:**
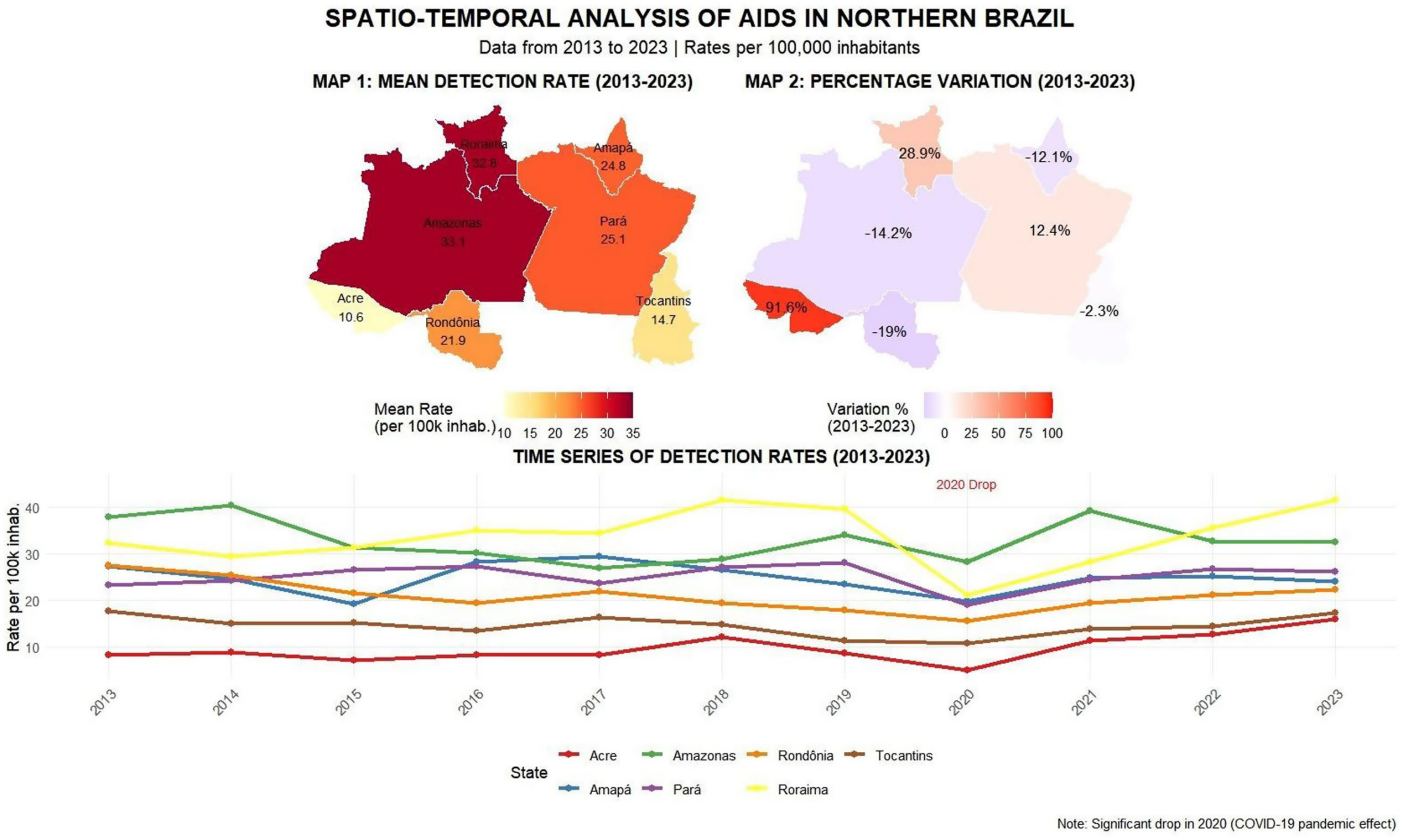
Spatial and temporal visualization of AIDS detection rates across the Northern region from 2013 to 2023. The left panel displays average detection rates per 100.000 inhabitants by state, while the right panel illustrates percentage variation over the decade. The time series below highlights annual fluctuations in detection rates across all federal units. Source: dos Anjos et al. (21).

Regarding the spatial analysis of detection rates from 2013 to 2023, Amazonas state shows the highest average AIDS rate at 33.1, followed by Roraima at 32.8; Pará at 25.1; Amapá at 24.8; and Rondônia at 21.9. In terms of rate variation, although Amazonas has the highest average rate, it showed a − 14.2% case reduction from 2013 to 2023. The greatest reduction occurred in Rondônia (−19%), followed by Amazonas (−14.2%), Amapá (−12.1%), and Tocantins (−2.3%). Conversely, Acre showed the most critical increase at 91.6%—higher than Pará’s 12.4% increase—while Roraima had the second highest increase at 28.9%. The line graph analysis revealed case reductions in all states during 2020, with Roraima standing out for maintaining high case rates throughout the entire 2013–2023 period, as described in Map 3.

**Map 4:**
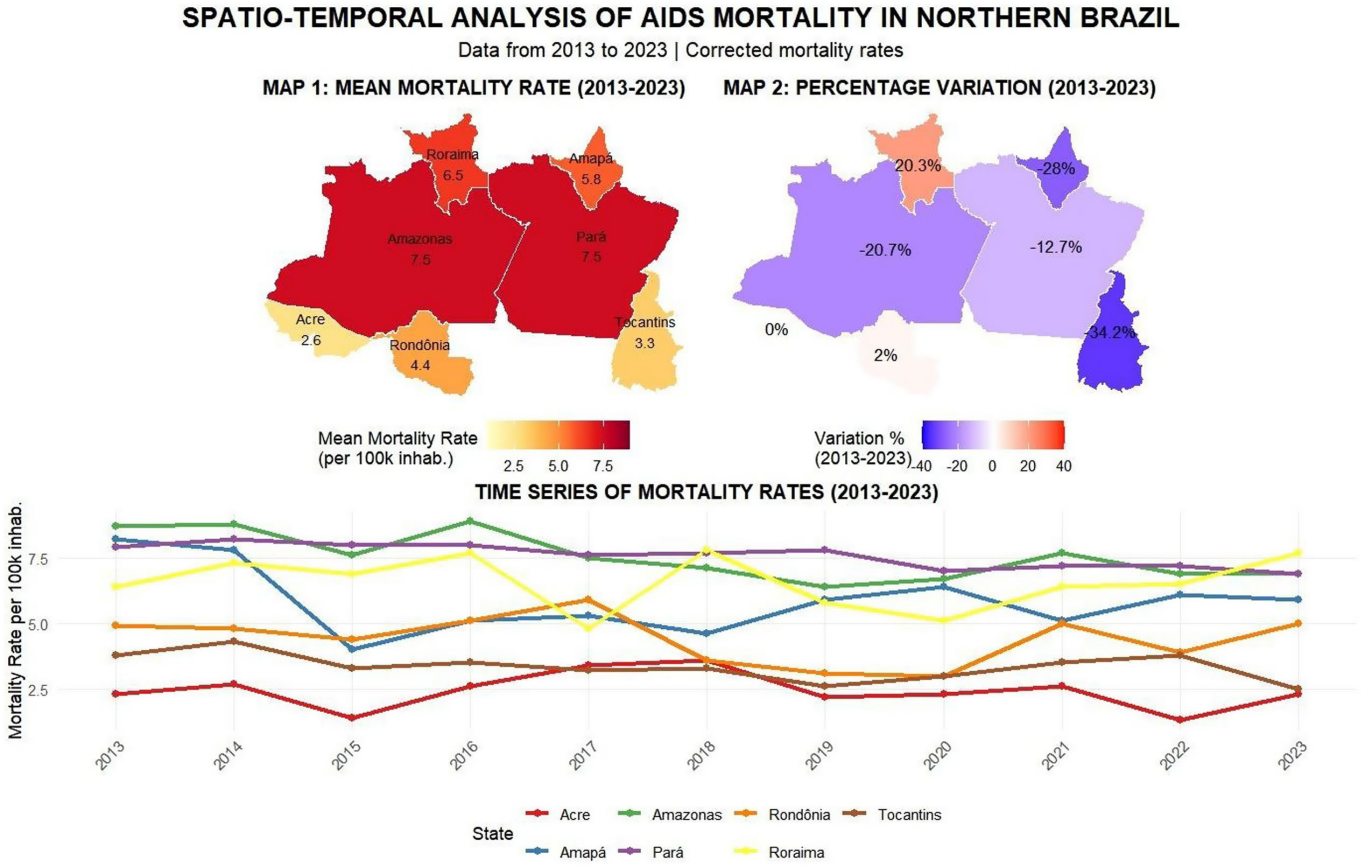
This panel presents a multi-layered view of AIDS mortality in Northern Brazil from 2013 to 2023. It includes: (1) a choropleth map showing average mortality rates by state; (2) a comparative map highlighting changes between the baseline year (2013) and 2023; and (3) line charts tracking annual variations in reported deaths over the decade. Source: dos Anjos et al. (21).

In mortality rates, Pará and Amazonas showed an average death rate of 7.5, followed by Roraima at 6.5, Amapá at 5.8, and Rondônia at 4.4. Regarding mortality variation, Tocantins showed the greatest reduction from 2013 to 2023 at −34.2%, followed by Amapá (−28%), Amazonas (−20.7%), and Pará (− 12.7%). Acre remained stable at 0%, while Roraima showed a 20.3% mortality increase and Rondônia 2%. The time series revealed significant fluctuations—in 2015, 4 of 7 federal units (Acre, Tocantins, Amapá, and Amazonas) showed case declines, while 2020 patterns differed across units: Roraima, Rondônia and Pará had notification declines, Acre remained stable, and Amapá/Amazonas increased. By 2023, new fluctuations emerged with Tocantins showing the steepest decline while Acre, Rondônia and Roraima increased, as shown in Map 4.

## 4 Discussion

This study evaluated confirmed AIDS cases and mortality in the Northern region of Brazil, the region with the largest territorial extension in Brazil, which has major socioeconomic gaps (8). In the description of cases, Amazonas, Pará, Rondônia and Tocantins stand out as having one of the highest numbers of cases and detection rates. It is worth noting that the federal units with the greatest vulnerability will probably have the greatest number of cases for late diagnosis, as well as aggravation for opportunistic diseases in the case of AIDS. In addition, it is observed that between 2019 and 2020, there was a decline in cases and detection rates of disease screening, this decrease must be related to the COVID-19 pandemic period, where Brazilian health services were overloaded and focused on COVID-19 health services (10–6). The states of Roraima and Amapá had higher detection rates than the North region and the Tocantins region. This increase can be partially explained by increased access to diagnostic, information, and prevention programs, such as pre- and post-exposure prophylaxis (PrEP and PEP), as well as reduced stigma and increased availability of testing. These measures have contributed to improved detection and increased case identification and reporting (10, 11).

Table 3 shows that mortality rates related to the disease were highest in Amazonas and Pará, higher than the rate for the Northern region, and that greater attention is needed in health actions to screen for AIDS cases. It is worth noting that the shortage of specialized services, the precariousness of education and prevention campaigns and late access to treatment all contribute to the development of opportunistic diseases and, consequently, to the high number of deaths from aids. In addition, the two states are the most populous in the North, which leads to greater notification compared to the others (12, 13). According to the studies by Sousa et al. (14), the northern region has been presenting actions that seek to achieve a greater mapping of the disease, with a view to reducing it. Our study found a significant reduction in mortality in Pará, Amazonas, Amapá and Tocantins. Factors linked to this include greater implementation and enforcement of HIV/aids public policies that include health programs, investments in structures, training and the provision of testing and treatment (13, 14).

In the violin graph, the analysis shows that the states with the highest concentrations of cases are Amazonas, Pará and Rondônia, with the greatest violin spacing over the 10-year period, the highest averages, medians and quartiles of cases, but still with a certain similarity to the Northern region, being the units that contribute most to the number of cases in the Northern region. In this sense, the identification of clusters and the mapping of disparities is essential in order to recognize areas that need assistance from the Ministry of Health’s health surveillance services to act to reduce cases so that this panorama changes over time, since Brazil aims to eliminate aids as a public health problem by 2030 (6–16). In this context, research mapping the disease is essential to find out whether health measures and public policies are being properly implemented and enforced to change the profile of the disease. This analysis is directly related to the study by Oliveira et al., where knowing the formulation, implementation and enforcement of public policies is essential to monitor the cycle of these policies through epidemiological data (17).

In Joinpoint’s analysis, the units showed distinct trends, with a green inflection point indicating changes in detection rates. Amapá, Amazonas, and Pará showed declines at this point, coinciding with the onset of the COVID-19 pandemic, a phenomenon referred to in this study as the “COVID-19 reporting decline.” The North region, Acre, Roraima, Tocantins, and Rondônia also showed reductions at the beginning of the pandemic; however, in subsequent years, the analysis revealed upward trends in these states. These changes are likely linked to the disruption of health services, reduced testing capacity, and the suspension of public awareness campaigns during 2020, which may have led to two distinct problems: underdiagnosis, due to reduced testing and limited access to care, and underreporting, resulting from delays or failures in reporting diagnosed cases (10–12). The increase in detection rates after 2020 may reflect the identification of previously undetected cases rather than a true increase in transmission. In addition to late detection, this increase may also be associated with the rise in risky sexual behaviors following the relaxation of social distancing measures and the discontinuation of prevention programs. In states like Roraima and Amapá, this increase may have several causes: both a real increase in new infections in the post-pandemic period and advances in access to testing and the resumption of prevention actions, which were developed to identify previously unreported cases (11). This scenario highlights the complexity of health service delivery and the need to assess how HIV/aids policies were implemented and adapted during the pandemic. Assessing the structural conditions, training, outreach, and investment in HIV/aids programs is essential, especially in the North, where disparities remain significant and directly influence case outcomes (17, 18).

In addition to the pandemic-related disruptions, it is important to consider the region’s specific characteristics that help contextualize this scenario. Although social and structural inequalities are present throughout Brazil, the North region has very unique characteristics that help explain why the incidence of aids remains high and, in some states, is even increasing. The combination of vast distances, low population density, and logistical challenges in accessing specialized health services directly affects early diagnosis and continued treatment. In states like Acre and Roraima, in addition to these factors, there are issues such as border dynamics, migration flows, and a health infrastructure that often fails to meet demand. These challenges hinder consistent prevention campaigns, the regular distribution of antiretrovirals, and the reach of isolated populations, such as indigenous and riverside communities. The increase in cases in the region should not be seen solely as a reflection of national inequities, but rather as a result of structural and logistical barriers specific to the region, which require strategies adapted to the local reality (10, 15, 18).

In heatmap 1, Amazonas and Roraima showed similar aids detection rates, while Pará, Amapá, and Rondônia showed similarities with the regional rate. This is concerning from an epidemiological perspective, as these states, in many years, exceed the detection rate of the reference point, the North region (6). The white and blue colors show stagnation and reduction in cases, indicating the effectiveness of health services in these areas, while the red units reflect persistently high rates between 2013 and 2023, revealing challenges that still need to be addressed (15). From this perspective, the heat map demonstrates the creation of clusters that help identify areas requiring targeted health interventions. These clusters are not random; they reflect underlying sociodemographic and structural similarities (12–14, 16–18). For example, Amazonas and Roraima share characteristics such as low population density, vast rural territories, and limited access to specialized health services, which contribute to similar epidemiological profiles. Similarly, the clustering of Pará, Amapá, and Rondônia with the regional average may be linked to shared public health challenges and the presence of vulnerable populations. This type of analysis is crucial in epidemiological studies, as it reveals whether significant changes are occurring by year and location, allowing for more accurate comparisons and guiding resource allocation (19).

Regarding the mortality rate in the linear regression, the North, Amazonas, and Pará showed a significant reduction, suggesting that the implementation of public policies and prevention efforts contributed to this result (12). However, other regions showed a weak and non-significant reduction, indicating that, despite progress in parts of the North, several states still require greater attention and reassessment of public health interventions (18). The Joinpoint model confirmed the downward trend in Tocantins, Amazonas, and Pará, with Pará showing a significant decline since 2014 (19). In contrast, Roraima showed an increase in deaths, raising concerns about the effectiveness of public policies, health education, availability of tests, and access to treatment and medication (12–14, 16–18). These findings should be interpreted in conjunction with the detection rate trends, as regions with higher detection rates and post-pandemic increases may reflect late diagnoses and limited access to care, which may contribute to higher mortality (20). The potential increase in deaths after 2022 in some regions may be associated with the lingering effects of the COVID-19 pandemic, including disruptions in treatment continuity, reduced health system capacity, and increased vulnerability among affected populations (18). These scenarios highlight the need for targeted government action to strengthen HIV/aids policies and reverse adverse trends.

The subsequent heatmap for mortality (Map 2) shows that although the North, Amazonas, and Pará experienced reductions in mortality rates, these variables continue to present the highest death rates, with the red areas and units clustered by similarity in the dendrogram representing zones requiring attention due to persistent challenges in mortality reduction (6–12, 15). The years marked in blue and white showed decreased mortality, potentially attributable to the effectiveness of public policies, as seen in Tocantins and Acre, which demonstrated mortality declines throughout the entire 2013–2023 period (13). In Map 3, although Amazonas, Rondônia, Amapá, and Tocantins exhibit high case averages, the percentage variation in detection rates indicates that these states’ efforts to reduce aids cases have been successful, as four of the seven units showed decreases from 2013 to 2023 (6–20). A key finding is Tocantins, which achieved a reduction despite Joinpoint initially indicating growth, demonstrating that while increases may occur, interventions to reduce cases are proving effective (13). The study highlights a concerning 90% increase in Acre, which poses a significant public health challenge for Brazil, as both aids and HIV cases are rising in the state (16). For mortality (Map 4), Amazonas and Pará showed similar average death rates, and again, four of the seven units achieved mortality reductions, reaffirming these states’ successful efforts to control aids as a public health issue (12–14, 16–18). However, Acre requires targeted actions and services, as its zero variation (stable situation) remains alarming, since it is unclear whether cases are declining or increasing (17–19).

As for the limitations of the study, one of the limiting factors was underreporting and lack of data, a factor that has been growing in recent years in one of the largest public record banks in Brazil, the Notifiable Diseases Information System (SINAN). Another limitation was the year, the researchers were going to analyze the year 2024, but due to registration bias because it was preliminary data, the study was between 2013 and 2023. Another potential limitation of this study is the use of 10-year averages in the analysis of spatial variation. While this approach is valuable for identifying long-term trends, it can obscure recent fluctuations in detection and mortality rates. For example, states like Roraima may appear favorable on average but show worsening indicators in more recent years. This highlights the importance of interpreting long-term data with caution and considering complementary analyses focused on recent periods to better capture current epidemiological dynamics. Our study focused on the 10-year trend, offering a broad and consolidated view. However, future studies that explore more recent data in a basic way will be essential to complement and deepen the results presented. Regarding the benefits of the research, the study provides details of all the federal units in the northern region of Brazil, the research presents new technologies and ways of analyzing data such as heatmap and Joinpoint, very few recent studies on aids trends and mortality were found, Those that were found did not cover the seven federative units and did not delve into mortality, so the research presents a trend study that in addition to mapping all the federative units, presents an in-depth analysis of aids and mortality in one of the regions with the greatest sociodemographic and economic vulnerabilities in Brazil.

## 5 Conclusion

Understanding the epidemiological situation of aids is crucial for disease control, particularly in regions with significant disparities. This study, conducted in Northern Brazil and its seven federal units, revealed a notable pattern of declining aids trends and mortality rates in the region, with four of the seven states showing reductions in both detection rates and mortality. A key finding was the 90% increase in detection rates in Acre and concerning mortality stabilization, which obscures whether cases are decreasing or increasing.

Another significant finding was the rise in cases shown in both heatmaps for Roraima—for both detection and mortality rates—highlighting the need for targeted health interventions to identify contributing factors. The percentage variation analysis showed increases exceeding 20% in both detection and mortality rates. A particularly noteworthy finding, not reported in previous studies, was the mortality rate reduction in Pará since 2014 and in Amazonas since 2019—especially significant as these are the most populous states in Northern Brazil, with Amazonas being Brazil’s largest state.

A research gap identified was the scarcity of trend studies analyzing federal units by local researchers, with most existing studies focusing on municipal-level data rather than state-wide analyses. This study encourages researchers in Northern Brazil and its federal units to contribute more studies mapping and clustering aids patterns in their states, as current literature focuses more on HIV than aids.

Effective implementation of programs, campaigns, testing, and treatment must form the foundation for aids reduction and control in these federal units. Given that aids remains a persistent challenge in several states, integrating epidemiological surveillance and prioritizing the Amazon region in HIV/aids response plans are essential pillars for reducing health disparities within the region itself.

## Data Availability

The datasets presented in this study can be found in online repositories. The names of the repository/repositories and accession number(s) can be found in the article/supplementary material.
